# On the estimation of population cause-specific mortality fractions from in-hospital deaths

**DOI:** 10.1186/s12916-019-1267-z

**Published:** 2019-02-08

**Authors:** Gail M. Williams, Ian Douglas Riley, Riley H. Hazard, Hafizur R. Chowhury, Nurul Alam, Peter Kim Streafield, Veronica Tallo, Diozele Sanvictores, Marilla Lucero, Tim Adair, Alan D. Lopez

**Affiliations:** 1 School of Public Health, University of Queensland, Brisbane, Australia; 2 School of Population and Global Health, University of Melbourne, Parkville, Australia; 3 International Centre for Diarrhoeal Disease Research, Dhaka, Bangladesh; 4 Research Institute for Tropical Medicine, Muntinlupa City, Philippines

**Keywords:** Bangladesh, Philippines, Vital registration, Cause of death, Death certificate

## Abstract

**Background:**

Almost all countries without complete vital registration systems have data on deaths collected by hospitals. However, these data have not been widely used to estimate cause of death (COD) patterns in populations because only a non-representative fraction of people in these countries die in health facilities. Methods that can exploit hospital mortality statistics to reliably estimate community COD patterns are required to strengthen the evidence base for disease and injury control programs. We propose a method that weights hospital-certified causes by the probability of death to estimate population cause-specific mortality fractions (CSMFs).

**Methods:**

We used an established verbal autopsy instrument (VAI) to collect data from hospital catchment areas in Chandpur and Comilla Districts, Bangladesh, and Bohol province, the Philippines, between 2011 and 2014, along with demographic covariates for each death. Hospital medical certificates of cause of death (death certificates) were collected and mapped to the corresponding cause categories of the VAI. Tariff 2.0 was used to assign a COD for community deaths. Logistic regression models were created for broad causes in each country to calculate the probability of in-hospital death, given a set of covariate values. The reweighted CSMFs for deaths in the hospital catchment population, represented by each hospital death, were calculated from the corresponding regression models.

**Results:**

We collected data on 4228 adult deaths in the Philippines and 3725 deaths in Bangladesh. Short time to hospital and education were consistently associated with in-hospital death in the Philippines and absence of a disability was consistently associated with in-hospital death in Bangladesh. Non-communicable diseases (excluding stroke) and stroke were the leading causes of death in both the Philippines (33.9%, 19.1%) and Bangladesh (46.1%, 21.1%) according to the reweighted method. The reweighted method generally estimated CSMFs that fell between those derived from hospitals and those diagnosed by Tariff 2.0.

**Conclusions:**

Statistical methods can be used to derive estimates of cause-specific probability of death in-hospital for Bangladesh and the Philippines to generate population CSMFs. In regions where hospital death certification is of reasonable quality and routine verbal autopsy is not applied, these estimates could be applied to generate cost-effective and robust CSMFs for the population.

**Electronic supplementary material:**

The online version of this article (10.1186/s12916-019-1267-z) contains supplementary material, which is available to authorized users.

## Background

Well-functioning vital registration (VR) systems are essential to monitor health progress and inform health policy. They are a critical input for public health analyses, understanding epidemiological patterns, and allocating scarce resources for public health and medical care. Most developed countries have complete or near complete VR systems, with reasonably accurate cause of death data. However, VR systems in low- and middle-income countries (LMICs) are generally incomplete in reporting causes of death, and the need for representative, quality data on leading causes of death is even greater [1].

Reports of hospital deaths are available from nearly all LMICs. In the absence of a comprehensive VR system, many countries use hospital deaths as the basis for their cause of death (COD) reporting and the identification of health priorities [2]. The advantage of hospital-based reporting of deaths is that hospital practitioners have the investigative capacity to discriminate between conditions with common sets of symptoms, although this does not necessarily mean that causes of hospital deaths are being accurately diagnosed [3, 4]. The disadvantage of using hospital data to extrapolate cause-specific mortality fractions (CSMFs) to whole populations is that hospital deaths are a biased sample of all deaths and thus are unlikely to accurately represent the distribution of deaths by cause in the population.

Murray et al. show that this selection bias associated with age and sex can, however, be addressed once the probability of a person of a particular age and sex dying in hospital from a particular disease or injury is known [5]. It is then possible to calculate the population-level distribution of deaths by cause, age, and sex. Put simply:

$$ {H}_{asj}={D}_{asj}\times {P}_{asj} $$

where *H*_*asj*_ is the number of deaths in hospital from age group *a*, sex *s*, and cause *j*; *D*_*asj*_ is the number of deaths in the population from age group *a*, sex *s*, and cause *j*; and *P*_*asj*_ is the proportion of all population deaths from age group *a*, sex *s*, and cause *j* which occur in hospital. We can therefore estimate *D*_*asj*_ by dividing *H*_*asj*_ by *P*_*asj*_. Summation of *D*_*asj*_ over *a* and *s* gives the estimated total number of population deaths from cause *j*, from which the population CSMF, the primary quantity of interest for guiding public policy, can be directly calculated.

This approach was applied to deaths in Mexican hospitals between 1998 and 2005 to estimate population CSMFs for the population [5]. VR during this period was estimated to be 90% complete in Mexico and 97% of deaths were certified by a physician [6]. As a result, *P*_*asj*_ could be calculated precisely given the high levels of VR completeness, and CSMF extrapolations could be validated against VR, assuming it is of reasonable diagnostic accuracy. The authors found that even when reducing the amount of VR data to calculate *P*_*asj*_ to 9%, the relative error between the true and estimated CSMF was only 12%. The key unknown affecting the generalizability of this approach, however, is the stability of *P*_*asj*_ under variable population circumstances.

The major problem with this method, however, is that *P*_*asj*_ can be difficult to determine. For example, a study in Malawi found that estimating the proportion of child deaths that occur in health facilities was complicated by potential overreporting of child deaths in-facility due to social stigma [7]. To address this issue, Murray et al. suggest using *P*_*asj*_ values of neighboring countries. While this approach may be attractive due to its relative simplicity, its scientific basis is questionable. Murray et al. also argue that *P*_*asj*_ varies depending on regional socioeconomic status and other factors, such as education. In their Mexico dataset, they showed that the proportion of deaths in-hospital due to HIV/AIDS ranged from about 0.35 for the least literate to 0.7 for the most literate populations.

As a solution to the variability of *P*_*asj*_ between regions and the difficulty in determining it, we propose an empirical methodology (as opposed to indirect approach used in Mexico by Murray et al.) that allows researchers to use hospitals deaths to predict population CSMFs while correcting for factors that affect place of death. We applied this approach to two sites in the Philippines and Bangladesh to ascertain if the method might be a valid basis to derive national COD estimates. We tested this hypothesis by comparing CSMFs derived from the method with population CSMFs derived from verbal autopsy (VA) using the Tariff 2.0 method and national Global Burden of Diseases (GBD) estimates [8].

## Methods

### Study overview

**Fig. 1:**
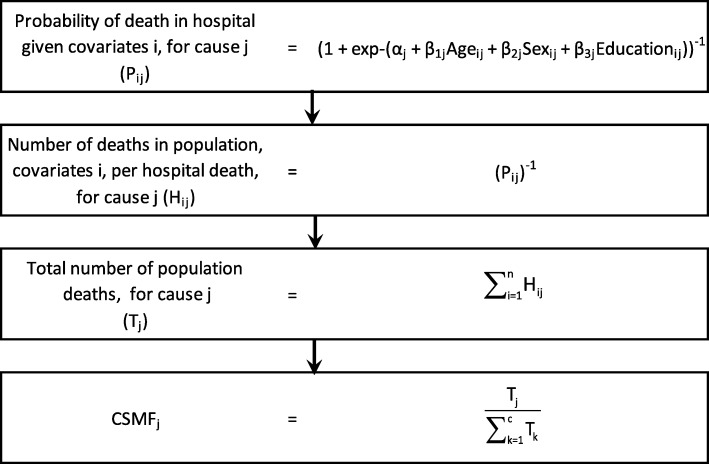
Process for calculating population cause-specific mortality fractions from verbal autopsy and hospital deaths with example covariates

Data were collected from hospitals and field sites in Chandpur and Comilla Districts in Bangladesh, and Bohol Province, Philippines, from 2011 to 2014. For each country, the Population Health Metrics Research Consortium (PHMRC) long form verbal autopsy instrument (VAI) was applied to a selection of community deaths in the hospital catchment area and Tariff 2.0 was used to assign a probable COD to each [8, 9]. Demographics, symptoms, and information on the place of death (hospital or out-of-hospital) for each death were collected from the VAI [10]. A set of covariates separate from the VAI (Additional file 1 and Additional file 2) were also collected for each death. For deaths that occurred in-hospital, a medical record review was conducted to establish, as confidently as possible, the true cause of death by applying the PHMRC “gold standard” diagnostic criteria. These gold standard criteria define ex ante standards for assigning causes of death using medical record review for the verbal autopsy cause list [9]. Figure 1 illustrates how this information was used to predict population CSMFs with a set of example covariates, where *i* represents a death in the hospital catchment area with place of death known and *k* represents a death that occurred in-hospital or out-of-hospital (COD assigned by Tariff 2.0) due to cause *j*. *α*, *β*_1_, *β*_2_, and *β*_3_, represent example parameter coefficients from a logistic regression model predicting probability of death in hospital, by cause.

Data were collected from each of the following two sites:

*Philippines*

Population VAs

Bohol is an island province with a population of about 1.2 million. It has 47 municipalities and one city (Tagbilaran City). Verbal autopsies were collected from all deaths in 11 of the municipalities which had been selected as clusters with probability of selection proportional to size. Deaths were identified by the capture-recapture method from three sources: the civil register, health center records, and the Catholic Church parish registers. These would be expected to represent 90–95% of all deaths [11].

Hospital death certificates

Death certificates were collected for all hospital deaths in Bohol Regional Hospital and the seven private hospitals in Tagbilaran City, the capital of the province. The catchment areas of these hospital covered the whole province (Additional file 3).

*Bangladesh*

Population VAs

VAs were collected from all deaths in the Matlab Subdistrict in Chandpur District with a total population of approximately 225,000. Rural populations in Chandpur and Comilla Districts are relatively homogeneous, and we considered the Matlab Subdistrict to be representative of the two districts.

Hospital death certificates

Death certificates were collected from eight public hospitals (one secondary level district hospital and seven subdistricts), six private hospitals (including the ICDDR,B research hospital), and Comilla Medical College Hospital (Additional file 4).

*Cause of death categorization*

For purposes of comparison, it was necessary to utilize common cause lists for hospital deaths and the VAI. The cause list was based on the GBD 2010 classification:

A. Communicable, maternal, neonatal, and nutritional conditions
B. Non-communicable diseases
C. Injuries

We divided these classifications into subgroups assuming different behaviors for different diseases based on our prior experiences with the functioning of health services in LMICs.

- *Group A*: Acute infections, chronic infections, and maternal conditions
- *Group B*. Diabetes and stroke were the two largest subgroups, followed by neoplasms. A large, heterogeneous group of non-communicable diseases (NCDs) remained, but we were limited in the number of disease groups we could use to establish the method.
- *Group C*. Injuries; we expected that these would receive priority for hospital care as a response to a recent acute episode.

The final broad cause groups are shown in Additional file 5.

*Verbal autopsy*

The PHMRC long form VAI was used to conduct VAs. In Bangladesh, the Matlab VAI was adapted to included PHMRC VAI question items given that Matlab has used and developed their own VA questionnaires since the late 1970s [12]. The VAs were conducted by community health workers following multiple team training programs. More information about the implementation of the VA data collection in Bangladesh can be found elsewhere [13]. Smart VA-Analyze was used to assign a COD using the Tariff 2.0 method for the population deaths [8].

*Hospital deaths*

It was necessary to code all hospital deaths irrespective of the quality of the clinical record or death certificate. To evaluate quality, clinical categories were established according to four different levels based on the criteria applied for the PHMRC gold standards [9]. These criteria classified deaths into four levels based on the degree to which the information from the medical record would provide sufficient certainty to determine whether the death could be used as part of the VA validation study. GS1, GS2A, and GS2B characterized cases where the medical records were of high quality; GS1 diagnoses provide the highest level of diagnostic certainty possible for that condition, consisting of either an appropriate laboratory test or X-ray with positive findings, as well as medically observed and documented illness signs. GS2A diagnoses are of a high level of diagnostic certainty, consisting of medically observed and documented illness signs. GS2B was developed for chronic conditions where the original records were not available but where records of treatment schedules were available from a reputable hospital. The PHMRC study, which was concerned with all deaths in hospital, introduced two more levels for categorizing cases where the cause of death information was of low quality. GS3 diagnoses related to medical or health worker diagnoses not supported by the appropriate level of investigation, but which met established clinical criteria, and GS4 diagnoses were cases unsupported by adequate clinical evidence. Cases classified as GS3 and GS4 were not considered suitable for inclusion in the PHMRC data base.

#### Demographic covariates

Covariates were collected for both the Bangladesh and Philippines sites using site-specific tools adapted to the local context (Additional file 1 and Additional file 2). These tools intended to collect variables that were associated with place of death and were readily available. A wealth index was computed using principal component analysis, based on a set of indicator or continuous variables describing household characteristics and assets (Additional file 6), based on the population sample of deaths. The factor loadings for the first principal component were used to calculate a wealth score for each death in the population sample. This process used PROC FACTOR and PROC SCORE in SAS 9.4. Quintile cut-offs were applied to scores to create five wealth categories. The same factor loadings and quintile cut-offs were then applied to hospital deaths.

#### Analysis

Causes of death in each country were stratified into broad groups based on GBD 2010 shown in Additional file 5. Within each stratum, multiple logistic regression was applied to the population sample to predict the probability of a death occurring in-hospital, given a set of covariate values. The reciprocal of this probability is the number of deaths (with the given covariate values) expected to occur in the population, for each such death in the hospital sample, for that COD stratum. This is referred to as the “predicted population deaths per hospital death” (DPHD).

Predicted deaths were summed for each COD to give an estimated total number of deaths in the population, now reweighted by inverse probability of occurring in hospital, from which CSMFs for the population were determined. Confidence intervals were calculated based on formulae derived from the precision of the logistic regression parameter estimates (Additional file 4).

A major issue which arises in applying this method is the choice of the covariate set to be used, which is potentially different for each COD, and which will influence the bias in, and precision of, the final CSMF estimates. To explore this further, a model selection process was identified as follows. An inclusive set of covariates for each COD was identified by preliminary analysis. This comprised the best six to eight predictors from univariate analyses. This was considered to be around the maximal number that could be included in a multivariable logistic regression using the “10 events per covariate” criterion to avoid overfitting [14]. All possible combinations of covariates up to the number in the maximal set were generated and logistic regression models fitted to each. These were then culled according to the following steps. Models which failed to converge to a solution or which had significant departure (*P* < 0.05) from goodness of fit (GOF) were discarded. Models were then ranked on three GOF criteria (Akaike information criterion (AIC), AUC, pseudo-r-squared). AIC is a measure of model goodness of fit, penalized for the number of parameters, AUC is a measure of the ability of a model to discriminate in and out-of-facility deaths, and pseudo-r-squared is a metric of explained variance for logistic regression models. The model with the best AIC was chosen for each COD.

CSMFs were also calculated without use of covariates (i.e., from the proportions of in-hospital deaths in the population sample for each COD). This method was termed “DPHD without (i.e., no) covariates” and denoted DPHDNC. For more details of the CSMF model estimators and confidence intervals, see Additional file 7.

The 2016 GBD cause of death estimates for the Philippines and Bangladesh were used as a comparator dataset [15]. The mapping between GBD level 3 causes and the broad cause categories is shown in Additional file 5.

## Results

We identified 4288 and 3725 adult deaths in the Philippines and Bangladesh, respectively. The proportion of deaths said to have occurred in-hospital from the VA interview varied substantially (Table 1). More deaths in the Philippines occurred in-hospital than in Bangladesh (Table 1). In the Philippines, a majority of maternal deaths occurred in-hospital, and a large portion of deaths due to stroke, other NCDs, acute infections, chronic infections, and injuries also occurred in-hospital. In Bangladesh, a large portion of maternal and injury deaths occurred in-hospital.

**Table 1:** Number (%) of deaths in hospital by site and cause

Site	All deaths	Cancer	Diabetes	Stroke	Other NCDs	Acute infections	Chronic infections	Maternal	Injuries
Philippines	942 (22.0)	72 (14.6)	42 (17.9)	197 (25.9)	353 (21.8)	105 (26.1)	31 (10.7)	19 (55.9)	123 (27.2)
Bangladesh	412 (11.1)	26 (7.0)	23 (9.9)	112 (9.6)	157 (12.2)	30 (9.9)	6 (9.4)	10 (35.7)	48 (18.0)

*ICD-10 codes for broad cause groupings shown in Additional file 5

The optimal model (best AIC) for each COD was approximately compliant with the 10 events per covariate guideline used to avoid overfitting. The covariates available for each cause varied depending on the number of deaths and model convergence. In both countries, age was consistently negatively associated with death in-hospital (Tables 2 and 3). In the Philippines, time to hospital less than 30 min was consistently the strongest predictor of death in-hospital. At least 8 years of education was also a strong predictor in the Philippines. Disability was a consistent predictor of death out-of-hospital in Bangladesh. Wealth, in terms of a wealth score or quintile derived from the list of available wealth-related covariates in Additional file 7 and PCA, was significantly associated with in-hospital death. However, given the logistical difficulties of deriving this value, wealth was excluded as a predictor in all other analyses. CSMFs using the wealth index are shown in Additional file 8.

**Table 2:** Philippines logistic regression models (Best AIC) by cause

Cause	Parameter	Age†	Sex‡	W/S/D	Education**	Occupation††	Time‡‡	Cost***	Intercept
Cancer	OR	0.75*	0.83*	–	1.20*	0.85	1.34*	1.08	–
	Beta coefficient	0.31	− 0.67	–	0.71	− 0.58	1.45	0.27	0.45
Diabetes	OR	0.73*	–	–	1.24*	0.82	1.57*	0.84	–
	Beta coefficient	− 0.39	–	–	0.84	− 0.74	1.95	− 0.62	0.74
Acute infections	OR	0.78*	–	0.89	1.12	0.85*	1.36*	1.06	–
	Beta coefficient	− 0.22	–	− 0.42	0.49	−0.58	1.42	0.23	0.17
Chronic infections	OR	0.71*	–	–	–	–	1.56*	1.62*	–
	Beta coefficient	− 0.41	–	–	–	–	2.33	1.78	−1.6
Injuries	OR	–	–	0.9	1.17*	0.90	1.38*	0.96	–
	Beta coefficient	–	–	−0.46	0.62	− 0.38	1.8	− 0.15	− 1.18
Maternal	OR	–	–	–	–	–	–	1.48	–
	Beta coefficient	–	–	–	–	–	–	1.44	− 0.59
Other NCDs	OR	0.80*	–	0.93	1.16*	0.95	1.43*	1.06	–
	Beta coefficient	− 0.24	–	−0.28	0.61	−0.2	1.7	0.22	− 0.34
Stroke	OR	0.80*	–	–	1.11	–	1.55*	1.09	–
	Beta coefficient	− 0.3	–	–	0.41	–	1.94	0.33	0.13

*Abbreviations*: *NCDs* non-communicable diseases, *W/S/D* widowed/separated/divorced

*(significant at the 0.05 level), † (decimal decades), ‡ (1 = Female, 2 = Male), ** (At least 8 years education), †† (Household occupation in agriculture or fishing), ‡‡ (Time to hospital ≤ 30 min), *** (Cost to hospital < 1000 Pesos)

**Table 3:** Bangladesh logistic regression models (Best AIC) by cause

Cause	Parameter	Age†	Sex‡	Disabled	Male HH	W/S/D	Municipality	Intercept
Cancer	OR	–	–	0.85	–	–	–	–
	Beta	–	–	− 0.64	–	–	–	− 2.39
Diabetes	OR	0.79	–	0.81	–	–	1.25	–
	Beta	− 0.37		− 0.77	–	–	0.92	0.3
Acute infections	OR	0.83	–	0.67*	–	0.66*	–	–
	Beta	− 0.21	–	− 1.5	–	−1.54	–	− 0.12
Chronic infections	OR	0.80	–	–	–	–	–	–
	Beta	− 0.31	–	–	–	–	–	− 0.32
Injuries	OR	–	–	0.47*	0.84	0.79	1.46*	–
	Beta	–	–	− 3.12	− 0.63	− 1.01	1.71	− 1.15
Maternal	OR	0.66	–	–	–	–	–	–
	Beta	− 0.89	–	–	–	–	–	1.94
Other NCDs	OR	0.79*	0.91	0.88*	–	0.84*	–	–
	Beta	− 0.3	− 0.37	− 0.49	–	− 0.68	–	0.75
Stroke	OR	0.87*	–	0.70*	–	–	–	–
	Beta	− 0.21	–	− 1.31	–	–	–	− 0.21

*Abbreviations*: *NCDs* non-communicable diseases, *HH* household, *W/S/D* widowed/separated/divorced, *OR* odds ratio, *CI* confidence interval

*(significant at the 0.05 level), † (decimal decades), ‡ (1 = Female, 2 = Male)

The results of applying the various methods to estimate community CSMFs in the Philippines are shown in Table 4. Other non-communicable diseases (NCDs), excluding stroke, was consistently the leading COD across the different estimation approaches, followed by stroke and acute infections. The fraction of deaths attributable to cancer, diabetes, chronic infections, and maternal deaths varied across methods. Maternal deaths clearly had the lowest CSMF, as might be expected, accounting for less than 5% of deaths.

**Table 4:** Philippines cause-specific mortality fraction (CSMF) comparison

Cause	CSMF	Hospital†	Deaths per hospital death model	Deaths per hospital death without covariates model	Community††	GBD‡
Cancer	Estimate 95% CI	3.9 (3.2,4.7)	6.9 (4.8,10)	6.1 (4.6,8.1)	11.5 (10.5,12.5)	12.7 (12.5–12.9)
Diabetes	Estimate 95% CI	3.8 (3.1,4.6)	4.7 (2.8,8)	4.8 (3.4,6.8)	5.5 (4.8,6.2)	4.3 (4.0,4.6)
Infections—acute	Estimate 95% CI	18 (16.5,19.5)	14.7 (11.6,18.6)	15.5 (12.9,18.8)	9.4 (8.5,10.2)	11.1 (10.8,11.3)
Infections—chronic	Estimate 95% CI	3.3 (2.6,4)	11.2 (4.7,26.8)	6.9 (4.6,10.2)	6.8 (6,7.5)	5.5 (5.2,5.9)
Injuries	Estimate 95% CI	11.8 (10.5,13.1)	8.9 (7.1,11.2)	9.8 (8.1,11.8)	10.6 (9.6,11.5)	7.7 (7.4,7.9)
Maternal	Estimate 95% CI	1.3 (0.9,1.8)	0.5 (0,0)	0.5 (0.3,0.8)	0.8 (0.5,1.1)	0.3 (0.3,0.3)
Stroke	Estimate 95% CI	20.7 (19.1,22.3)	19.1 (15.8,23.1)	18 (15.6,20.9)	17.8 (16.6,18.9)	11.9 (11.1,12.7)
Other NCDs	Estimate 95% CI	37.1 (35.2,39)	33.9 (29.2,39.3)	38.3 (34.2,42.9)	37.8 (36.3,39.2)	46.5 (45.9,47.1)

*Abbreviations*: *CI* confidence interval, *NCD* non-communicable diseases

† (cause assigned by medical record review), †† (cause assigned by Tariff 2.0), ‡ (2016 Global Burden of Disease Philippines national estimate)

Community CSMFs, assigned by the Tariff 2.0 VA method, and hospital CSMFs, assigned by medical record review, were similar for injury, maternal, and other NCD deaths. The CSMFs estimated from the DPHD method that considered covariates related to the probability of death in hospital were greater than the hospital estimates for cancer and chronic infection deaths. Chronic infection from the DPHD method had a wide confidence interval, likely due to the low probability of hospitalization for chronic disease deaths. The DPHDNC method estimates, which did not consider covariates related to death in-hospital, were similar to the estimates from the method with covariates, with the only notable exception being that the DPHDNC method estimated more other NCD deaths.

The national GBD estimates showed closest similarity with the community estimates, with the exception of injuries, stroke, and other NCDs. The community estimated more injury and stroke deaths while GBD estimated more other NCD deaths. Based on our experience with implementing the Tariff 2.0 method in several countries, a potential explanation for the large difference between community and GBD estimates for other NCDs and stroke could be incorrect translation or misinterpretation of the word “paralysis” in the VA instrument, leading to overreporting of paralysis and therefore a disproportionate number of stroke deaths predicted by Tariff 2.0.

Comparison of CSMFs from application of the same methods to Bangladesh data is shown in Table 5. Other NCDs and stroke were the leading COD across all methods. Other causes displayed moderate variation across the methods. In particular, while community and hospital estimates were similar for acute and chronic infections, community CSMF estimates were greater than the hospital estimates for cancer, diabetes, and stroke deaths and less than the hospital estimates for injury, maternal, and other NCD deaths. The DPHD method estimates were generally between the community and hospital estimates. Again, the GBD estimates were closest to the community estimates, with community estimating more stroke deaths and GBD estimating more other NCD deaths, potentially for the reason as described above. The DPHDNC method estimates were similar to the estimates from the DPHD method, with the only notable exception being that the method without covariates estimated more injury deaths. Overall, estimates across all methods in Bangladesh displayed general agreement when considering the confidence intervals.

**Table 5:** Bangladesh cause-specific mortality fraction (CSMF) comparison

Cause	CSMF	Hospital†	Deaths per hospital death model	Deaths per hospital death without covariates model	Community††	GBD‡
Cancer	Estimate 95% CI	3.4 (2.5,4.2)	5.3 (3.6,7.9)	5.5 (3.5,8.7)	9.9 (8.9,10.9)	11.9 (11.6,12.2)
Diabetes	Estimate 95% CI	2.8 (2,3.6)	2.6 (1.7,4.1)	3.3 (2,5.3)	6.2 (5.5,7)	4.2 (3.9,4.6)
Infections—acute	Estimate 95% CI	9 (7.6,10.4)	10.4 (5.8,18.6)	10.4 (7.2,15.2)	8.1 (7.3,9.0)	8.6 (8.2,8.9)
Infections—chronic	Estimate 95% CI	2.1 (1.4,2.8)	2.1 (0.9,4.5)	2.6 (1.1,6.0)	1.7 (1.3,2.1)	2.6 (2.3,2.8)
Injuries	Estimate 95% CI	9.4 (7.9,10.8)	11.1 (4.7,26.1)	6 (4.4,8.1)	7.1 (6.3,8)	8.3 (8.0,8.6)
Maternal	Estimate 95% CI	3.9 (3,4.9)	1.4 (1.4,1.4)	1.3 (0.7,2.2)	0.8 (0.5,1.0)	0.6 (0.6,0.7)
Stroke	Estimate 95% CI	19.8 (17.8,21.7)	21.1 (17.0,26.1)	23.8 (19.4,29.3)	31.5 (30.0,32.9)	17 (16.1,17.9)
Other NCDs	Estimate 95% CI	49.7 (47.2,52.1)	46.1 (39,54.6)	47.1 (40.1,55.4)	34.7 (33.1,36.2)	46.8 (46.1,47.5)

*Abbreviations*: *CI* confidence interval, *NCD* non-communicable diseases

† (cause assigned by medical record review), †† (cause assigned by Tariff 2.0), ‡ (2016 Global Burden of Disease Bangladesh national estimate)

### Generalizability of the method

**Fig. 2:**
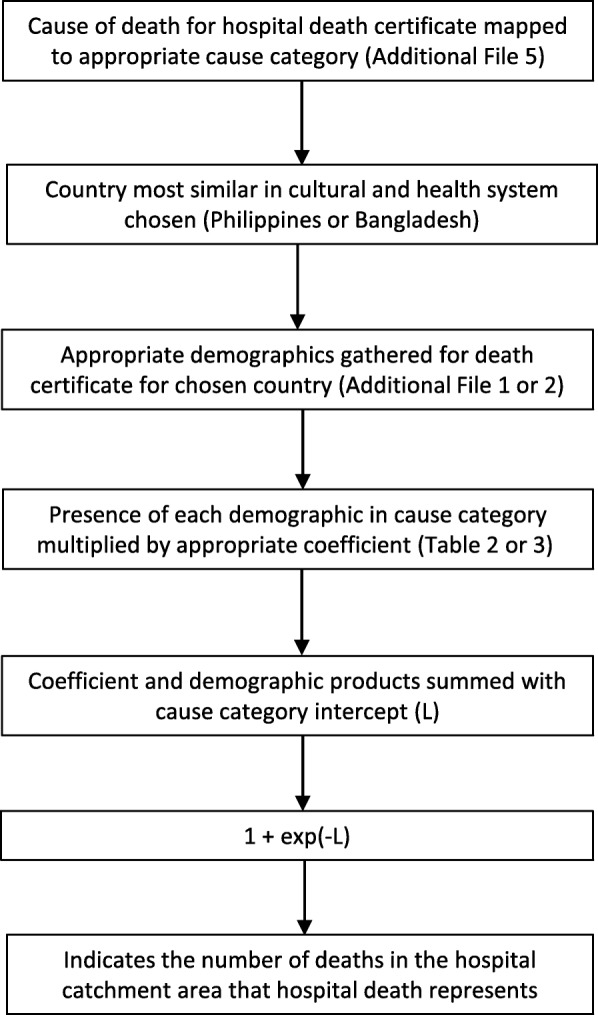
Process of using deaths per hospital death method

With appropriate prudence, the DPHD method proposed here could be applied in neighboring countries with approximately similar epidemiological and development characteristics to the Philippines and Bangladesh in order to estimate community COD patterns by inputting the beta coefficients indicated in Table 2 or Table 3 from Bangladesh or the Philippines. The choice of coefficients would depend on which country was judged to have the most similar proportion of deaths in-hospital, spatial dispersion of health care centers, and GDP per capita, among other factors, to the population for which estimates are required, or, perhaps more prudently, by using a combination of the two country models. The results may well be useful for developing a preliminary description of the cause of death pattern for community deaths, but in all cases should be revised on the basis of other epidemiological data and information about causes of death in that community. The coefficient values are inputted along with the appropriate covariate values from each individual medical record. In this way, and using a widely available tool such as Microsoft Excel, any hospital with reasonable diagnostic capacity can use this information to estimate the probable number of deaths that occur from different causes in the hospital catchment area. Figure 2 illustrates how this process could be routinely applied to estimate community CSMFs from hospital cause of death data. For example, if Bangladesh were chosen to be most similar to a given country in terms of culture and health care systems, and it was known with confidence that a 60-year-old man without a disability died of a stroke in-hospital, then that one in-hospital stroke death represents

$$ 1+{e}^{-\left(-0.21(6)-1.31(0)-0.21\right)}=5.3\ \mathrm{deaths}\ \mathrm{in}\ \mathrm{the}\ \mathrm{hospital}\ \mathrm{catchment}\ \mathrm{area}. $$

Systematic application of the cause-specific coefficients from Bangladesh would yield a probable cause of death distribution for the population served by that hospital.

## Discussion

We have proposed a novel method to estimate cause-specific mortality patterns from in-hospital mortality statistics, which are generally available in most low- and middle-income countries. The method builds upon other approaches that adjust hospital mortality data for the population by taking an empiric, rather than indirect, approach and offering a simple application to hospital catchment areas. Application of this method identified stroke and other NCDs as the most frequent COD in the Philippines and Bangladesh. Hospital death certificates, as well as application of Tariff 2.0 to the hospital catchment area, also identified these two causes as the most frequent COD. The rank order of importance of the remaining causes of death varied across the methods under comparison, although the DPHD method estimates generally estimated a CSMF somewhere between that resulting from hospital data and the community. The CSMFs from the models with and without covariates were similar across all causes in both populations. Weighting by the probability of a particular cause occurring in-hospital potentially adjusted for the overrepresentation of causes that are more likely to occur in-hospital, such as maternal deaths, and the underrepresentation of causes that are more likely to occur at home, such as cancer deaths.

The likelihood of a person dying in-hospital is affected not only by age, sex, and cause, but also by demographic factors relating to the culture and health care systems of the deceased [5]. Therefore, simply using the probability of death in-hospital from neighboring countries is unlikely to produce accurate cause of death estimates. The variability in the proportion of deaths in-hospital between the Philippines and Bangladesh demonstrates the difficulty in using the values of neighboring countries. Consequently, we adjusted for covariates (Additional files 1 and 2) that influence place of death, making the results potentially more applicable across countries. Our findings suggest that distance from hospital, marital status, and wealth influence the place of death. We found that time to hospital was the greatest predictor of death occurring in-hospital in the Philippines, while presence or absence of a disability was the greatest predictor of death out-of-hospital in Bangladesh. Wealth was a strong predictor of death in hospital in Bangladesh, but we were unable to use wealth in the final analysis due to the difficulty in determining wealth from medical records. Models that include wealth as a score or a quintile, or a binary variable indicating TV or fridge ownership are shown in Additional files 7 and 8.

In addition to the DPHD method that adjusts for covariates, we also estimated CSMFs without adjusting for covariates by simply using the proportion of people who died in-hospital for each cause according to Tariff 2.0 or medical record review. We found the CSMFs from this approach were similar to the method using covariates. While weighting by the probability of dying in-hospital without using covariates is far simpler than collecting the appropriate covariates for each hospital death, it is not possible for other countries or regions to use this method without conducting VAs on the hospital catchment area. Thus, adopting this estimation approach negates the time and cost savings of applying this methodology elsewhere.

Unfortunately, it is not possible to validate the DPHD method as neither Bangladesh nor the Philippines have complete VR systems. However, our methodology follows the work of Murray et al., who demonstrated that even in areas in Mexico with reduced VR coverage, hospital deaths weighted by the proportion of deaths in-hospital produced more accurate population CSMFs than hospital deaths alone [5]. We might therefore infer that the DPHD method in the Philippines and Bangladesh produced more accurate cause of death estimates than the hospital CSMFs.

It is difficult to identify an appropriate set of validation data against which to test the predictive performance of our approach to estimating cause of death patterns. Probably the most appropriate comparator data set are the country-level GBD estimates since they make extensive use of all available mortality and covariate data, appropriately adjusted for known biases and modeled to describe the likely COD patterns in 195 countries [15]. Since the GBD study does not provide sub-national disease estimates for the Philippines or Bangladesh, we do not have a better validation dataset for the COD distribution in study sites. We found that the GBD showed general agreement with the DPHD method but showed closer agreement with the community results. We cannot determine the relative performance of the DPHD method versus community, but we can assert that the method can be far more easily applied than conducting VAs. As VR systems improve, the implied cause distribution in the population might be compared with that of the DPHD method as one external measure of plausibility.

While the DPHD method produces less-biased estimates than hospital deaths alone and can be more easily applied than VA, there are implementation issues that could affect the validity of estimates. First, we attempted to use demographic covariates, such as age, sex, marital status, and education, that are widely available in hospital medical records and would be influential in determining place of death, but some countries may have difficulty collecting this information in a systematic fashion or the covariates may be unavailable. Without these covariates, population CSMFs cannot be calculated. Second, a recent systematic review questioned the quality of hospital diagnoses, which for this method has assumed to be a “gold standard” [16]. Poor hospital diagnoses would bias the extrapolation to the hospital catchment area. Similarly, VA was used to assign individual causes to community deaths. VA is the only plausible way to assign causes to these deaths and has reasonable performance at the population level, but less so at the individual-cause level [8]. However, as discussed earlier, some of the differences between the community and GBD estimates for various causes might well be attributable to misinterpretation of the VA questionnaire by the respondents. Third, to consider the international variations in diagnostic practices, capacity, and skills, we condensed COD into eight broad causes, which limits the granularity of extrapolated CSMFs. Nonetheless, the broad cause categories that we have proposed still provide valuable information on the likely stage of the epidemiological transition in the population to which it is applied. Future research, where possible, should attempt to use more specific causes of death. In addition, future studies should consider the difference in diagnostic accuracy between hospital death certification and VA, which we did not take into account in this study. Fourth, while we could calculate confidence intervals for the modeled CSMFs in Bangladesh and the Philippines, this would not be possible in other countries without the variance-covariance matrix of the beta coefficients, which necessitates data from a population survey. However, model confidence intervals may be less of a limitation given recent controversy over the use of confidence intervals in global health research, given the fact that variability is likely to be at least as much affected by other factors related to data quality as model specification [17]. Finally, no true validation dataset exists in which to validate the DPHD method. We attempted to use the most available and robust data to assess its performance. Given the limitations of the data, we were unable to test whether the method could be used within or between countries. The Philippines and Bangladesh have relatively similar mortality profiles. Future research should attempt to validate this method against data sources for other countries with somewhat different mortality profiles. Beyond the scope of this paper, there are potentially other data that could be used to test the performance of the proposed method, such as patterns of age-specific death rates as well as application of the method to other countries without complete VR systems but with readily available hospital and census data, such as Brazil and Ecuador [18, 19].

## Conclusion

Informed health policy requires reliable estimates of the leading causes of death for the entire population, not just for those who die in hospital given that the probability of dying in-hospital is likely to be age, cause, sex, and culturally dependent. Hospital CSMFs need to be weighted by the region and cause-specific probability of a death occurring in-hospital. It is possible to estimate these probabilities and use cause-specific logistic regression models to estimate the cause-specific probability of a death occurring in-hospital. These regression models could potentially extrapolate hospital CSMFs to the population in regions that are deemed to be culturally similar to the Philippines or Bangladesh, the two countries on which the method has been developed. For regions and countries that have not employed routine VA, and where deaths in-hospital are certified with reasonable accuracy, information collected from routine hospital medical records and death certificates can be used to generate, at no, or very low, cost, reasonably plausible population CSMFs.

## Additional files

Additional file 1: Philippines covariates form. Form used to collect demographic demographics of the decreased in the Philippines. (PDF 84 kb)

Additional file 2: Bangladesh covariates form. Form used to collect demographics covariates of the decreased in Bangladesh. (PDF 49 kb)

Additional file 3: Map of Bohol, Philippines. Map of hospital catchment areas in Bohol. (PNG 6975 kb)

Additional file 4: Map of Bangladesh. Map of hospital catchment areas in Bangladesh. (PNG 404 kb)

Additional file 5: Cause of death map. Cause of death mapping between the broad cause categories, Tariff 2.0 cause categories, and Global Burden of Disease cause categories. (XLSX 19 kb)

Additional file 6: Death per hospital death model appendix. Additional information on calculating the point estimates and confidence intervals for the deaths per hospital death model. (DOCX 16 kb)

Additional file 7: Bangladesh and Philippines wealth index calculation. Demographic covariates used to calculate a wealth index using principal component analysis (PCA) for Bangladesh and the Philippines. (XLSX 13 kb)

Additional file 8: Bangladesh and Philippines cause-specific mortality fractions for death with wealth variables. Cause-specific mortality fractions for the death per hospital death model with wealth score, wealth quintile, wealth indicator, or no wealth. (XLSX 12 kb)

## Abbreviations

AIC: Akaike information criterion
AUC: Area under the curve
CI: Confidence interval
COD: Cause of death
CSMF: Cause-specific mortality fraction
DPHD: Death per hospital death
DPHDNC: Death per hospital death without covariates
GBD: Global Burden of Disease
GOF: Goodness of fit
LMICs: Low and middle-income countries
NCD: Non-communicable diseases
PCA: Principal component analysis
PHMRC: Population Health Metrics Research Consortium
VA: Verbal autopsy
VAI: Verbal autopsy instrument
VR: Vital registration
