# Supplementary material for: On the estimation of population cause-specific mortality fractions from in-hospital deaths
Source: BMC Med. 2019 Feb 8;17:29. doi: 10.1186/s12916-019-1267-z (PMC6367755; doi:10.1186/s12916-019-1267-z)
Supplement: Supplementary file 1 — Philippines covariates form. Form used to collect demographic demographics of the decreased in the Philippines. (PDF 84 kb) [file 12916_2019_1267_MOESM1_ESM.pdf]

# CAUSE OF DEATH (COD) STUDY

## Covariates of Death Events

## 1. INFORMATION ABOUT THE DECEASED

1.1. Name of the Deceased ____________________________ ______________________
Last Name First name

1.2. Date of Birth |___||___| |___||___| |___||___||___||___|
dd mm yyyy

1.3. Date of Death |___||___| |___||___| |___||___||___||___|
dd mm yyyy

1.4 Sex |___|
|___| 1 Male
|___| 2 Female

1.5. Marital status (for deceased 10 yrs old and above) |___|
|___| 1 Never married
|___| 2 Married
|___| 3 Widowed/separated

1.6. Religion |___|
|___| 1 Roman Catholic
|___| 2 Protestant
|___| 3 Christian
|___| 4 Moslem
|___| 5 Others, specify_______________
____________________________

1.7. Usual place of residence ___________________________________ |___||___|

1.8. Municipality of residence at terminal illness ___________________________________

1.9. Municipality of residence at death ___________________________________

1.10. Number of years of schooling completed Verbatim ________________________ |___||___|

1.11. Occupation at time of death (Most recent occupation) for deceased more than 10 years old, pls specify ____________________________________________________ |___||___|

1.12. Name of Mother (if deceased is <12 yrs old) ____________________________ ______________________
Last Name First name

1.13. Date of Birth of Mother |___||___| |___||___| |___||___||___||___|
dd mm yyyy

1.14. Marital status of Mother |___|
|___| 1 Never married
|___| 2 Married
|___| 3 Widowed/separated

1.15. Number of years of schooling completed **VERBATIM** _________________________ |___||___|

1.16. Occupation , pls specify ____________________________________________________ |___||___|

1.17. Name of Father
(if deceased is <12 yrs old)

Last Name First name

1.18. Date of Birth |___||___| |___||___| |___||___||___||___|
dd mm yyyy

1.19. Number of years of schooling completed **VERBATIM** __________________________ |___||___|

1.20. Occupation , pls specify __________________________________________________ |___||___|

## 2. INFORMATION ABOUT THE HOUSEHOLD HEAD

2.1. Name of the HH head ___________________________ ___________________________
Last Name First name

2.2. Date of Birth |___||___| |___||___| |___||___||___||___|
dd mm yyyy

2.3. Sex |___| 1 Male |___|
|___| 2 Female

2.4. Marital status |___| 1 Never married |___|
|___| 2 Married
|___| 3 Widowed/separated

2.5. Number of years of schooling completed _________________________________ |___||___|

2.6. Occupation , pls specify _________________________________________________ |___||___|

## 3. HOUSEHOLD ASSETS

3.1. Does any household member have a health insurance, e.g. PhilHealth, MediCard, CARITAS? |___| 1 Yes |___| 2 No |___|

3.2. Number of HH members **ERBATIM** ___________________________ |___||___|

3.3. Number of sleeping rooms **VERBATIM** ___________________________ |___|

3.4. Source of drinking water |___|
|___| 1 Rain
|___| 2 Spring
|___| 3 Pond/River/Stream
|___| 4 Public unprotected well
|___| 5 Public protected well
|___| 6 Household unprotected well
|___| 7 Household protected well
|___| 8 Public tap water
|___| 9 Neighbor's tap water
|___|10 Tap water inside the house
|___| 11 Bottled

3.5. Toilet type |___|
|___| 0 None
|___| 1 Antipolo type

| | | | | |
|---|---|---|---|---|
| | | \|___\| 2. Water sealed<br>\|___\| 3 Flush type | | |
| 3.6. | Fuel used for cooking | \|___\| 0 Sawdust<br>\|___\| 1 Charcoal/Coconut husk<br>\|___\| 2. Firewood<br>\|___\| 3 LPG gas<br>\|___\| 4 Electricity | | \|___\| |
| 3.7. | Separate cooking room ? | \|___\| 1 Yes | \|___\| 2 No | \|___\| |
| 3.8. | Housing materials: floor | \|___\| 0 Earth/mid/sand<br>\|___\| 1 Bamboo<br>\|___\| 2. Wood<br>\|___\| 3 Cement/Tile | | \|___\| |
| 3.9. | Housing material: roof | \|___\| 0 Scrap material/sack/boxes<br>\|___\| 1 Thatch/nipa<br>\|___\| 2. GI roofing<br>\|___\| 3 Cement | | |
| 3.10. | Housing material: inner wall | \|___\| 0 Thatch/leaves<br>\|___\| 1 Bamboo/sawali<br>\|___\| 2. Wood<br>\|___\| 3 Cement/Tile | | |
| 3.11. | Have electricity ? | \|___\| 1 Yes | \|___\| 2 No | \|___\| |
| 3.12. | Own radio ? | \|___\| 1 Yes | \|___\| 2 No | \|___\| |
| 3.13. | Own television ? | \|___\| 1 Yes | \|___\| 2 No | \|___\| |
| 3.14. | Own a cd/vcd/dvd player ? | \|___\| 1 Yes | \|___\| 2 No | \|___\| |
| 3.15. | Own a component or karaoke? | \|___\| 1 Yes | \|___\| 2 No | \|___\| |
| 3.16. | Own refrigerator ? | \|___\| 1 Yes | \|___\| 2 No | \|___\| |
| 3.17. | Own a washing machine ? | \|___\| 1 Yes | \|___\| 2 No | \|___\| |
| 3.18. | Own a computer ? | \|___\| 1 Yes | \|___\| 2 No | \|___\| |
| 3.19. | Own landline fone ? | \|___\| 1 Yes | \|___\| 2 No | \|___\| |
| 3.20. | Own mobile fone ? | \|___\| 1 Yes | \|___\| 2 No | \|___\| |
| 3.21. | Own a bicycle ? | \|___\| 1 Yes | \|___\| 2 No | \|___\| |
| 3.22 | Own a motorcycle ? | \|___\| 1 Yes | \|___\| 2 No | \|___\| |
| 3.23. | Own a car ? | \|___\| 1 Yes | \|___\| 2 No | \|___\| |
| 3.24. | Own a motorboat ? | \|___\| 1 Yes | \|___\| 2 No | \|___\| |

**4. DISTANCE AND TRAVEL TIME TO BRH (TO BE ASKED ONLY IF DEATHS TOOK PLACE AT BRH; OTHERWISE PROCEED TO SECTION 5.)**

| | | | |
|---|---|---|---|
| 4.1. | Distance of residence to BRH in kilometers (if residence is outside the poblacion, get the distance of the barangay from the poblacion) information on distance will be validated from existing information. | | \|___\|\|___\|\|___\| |
| 4.2. | Did you bring the deceased directly to BRH? | \|___\| 1 Yes **CONTINUE** \|___\| 2 No **SKIP TO 4.5** | \|___\| |
| 4.3. | If Yes, how long did you have to travel from your house to BRH (in minutes) ? | **VERBATIM** ________________ | \|___\|\|___\|\|___\| |
| 4.4. | How much did you spend to travel to BRH ? | **VERBATIM** ________________ | \|___\|\|___\|\|___\|\|___\| |

**TERMINATE INTERVIEW**

| | | | |
|---|---|---|---|
| 4.5. | If NO, to which hospital, health facility or physician, did you bring her/him to **FIRST** before s/he was moved to BRH? | **VERBATIM** ________________ | \|___\|\|___\| |
| 4.6 | How long did you have to travel from your house to this facility in minutes ? | **VERBATIM** ________________ | \|___\|\|___\|\|___\| |
| 4.7. | How much did it cost you to travel from your house to this facility ? | **VERBATIM** ________________ | \|___\|\|___\|\|___\|\|___\| |
| 4.8. | How long did you have to travel from this facility to BRH ? | **VERBATIM** ________________ | \|___\|\|___\|\|___\| |
| 4.9. | How much did it cost you to travel from this facility to BRH ? | **VERBATIM** ________________ | \|___\|\|___\|\|___\|\|___\| |

**TERMINATE INTERVIEW**

**5. DISTANCE AND TRAVEL TIME TO NEAREST HOSPITAL AND BRH (TO BE ASKED OF ALL DEATH EVENTS WHICH DID NOT TAKE PLACE IN BRH)**

| | | | |
|---|---|---|---|
| 5.1. | Where the deceased die ? | \|___\| 1 Home **(CONTINUE)**<br>\|___\| 2 Hospital, name of hospital ____________________________ **SKIP TO Q 5.4.**<br>\|___\| 3 On the road **SKIP TO Q.5.10.**<br>\|___\| 4 Others,____________________________ **SKIP TO Q 5.10.** | \|___\| |

| | | | |
|---|---|---|---|
| 5.2. | If you were to bring the deceased to BRH, how long would it take you to go there (in minutes)? | **VERBATIM** ____________________ | |___||___||___| |
| 5.3. | How much would it cost you to go there ? | **VERBATIM** ____________________ | |___||___||___||___| |

**TERMINATE INTERVIEW**

| | | | |
|---|---|---|---|
| 5.4. | Did you bring the deceased directly to this facility? | |___| 1 Yes **CONTINUE**    |___| 2 No **SKIP TO 5.7.** | |___| |
| 5.5. | How long did you have to travel from your house to this facility (in minutes )? | **VERBATIM** ______________________ | |___||___||___| |
| 5.6. | How much did it cost you to travel to this facility ? | **VERBATIM** ______________________ | |___||___||___||___| |

**TERMINATE INTERVIEW**

| | | | |
|---|---|---|---|
| 5.7. | If NO, to which hospital, health facility or physician, did you bring her/him to **FIRST** before s/he was brought to the facility where s/he died ? | **VERBATIM** ______________________ | |___||___| |
| 5.6. | How long did you have to travel from your house to this facility (in minutes) ? | **VERBATIM** ______________________ | |___||___||___| |
| 5.7. | How much did it cost you to travel from your house to this facility ? | **VERBATIM** ______________________ | |___||___||___||___| |
| 5.8. | How long did you have to travel from this facility to the facility where the deceased died ? | **VERBATIM** ______________________ | |___||___||___|| |
| 5.9. | How much did it cost you to travel from the first facility to the facility where the deceased died ? | **VERBATIM** ______________________ | |___||___||___||___| |

**TERMINATE INTERVIEW**

| | | | |
|---|---|---|---|
| 5.10. | Where did you plan to bring the deceased then ? | **VERBATIM** ______________________ | |___||___| |
| 5.11. | How long would you have to travel to get there (in minutes) ? | **VERBATIM** ______________________ | |___||___||___| |

| | | | |
|---|---|---|---|
| 5.12. | How much would it have cost you to get there (in minutes) ? | **VERBATIM** ______________________ | |___||___||___||___| |
| 5.13. | If you were to bring the deceased to BRH, how long would it take you to go there (in minutes)? | **VERBATIM** ______________________ | |___||___||___| |
| 5.14. | How much would it cost you to go there ? | **VERBATIM** ______________________ | |___||___||___||___| |

**TERMINATE INTERVIEW**

Name of Data Abstractor ________________________________

Date of Household Visit |___||___| |___||___| |___||___||___||___|
dd mm yyyy
