# Supplementary material for: On the estimation of population cause-specific mortality fractions from in-hospital deaths
Source: BMC Med. 2019 Feb 8;17:29. doi: 10.1186/s12916-019-1267-z (PMC6367755; doi:10.1186/s12916-019-1267-z)
Supplement: Supplementary file 2 — Bangladesh covariates form. Form used to collect demographics covariates of the decreased in Bangladesh. (PDF 49 kb) [file 12916_2019_1267_MOESM2_ESM.pdf]

|___||___||___||___|

CAUSE OF DEATH (COD) STUDY
COVARIATES INFORMATION OF DEATHS
CHANDPUR DISTRICT, BANGLADESH

1. INFORMATION ABOUT THE DECEASED

Upazila: _______________ Village/Moholla: _______________ Bari/Holding#______

1.1	Name of the deceased		RID:<br>CID:
1.2	Date of death		|___||___| |___||___| |___||___||___||___| dd mm yyyy
1.3	Date of birth: Age ______ years		|___||___| |___||___| |___||___||___||___| dd mm yyyy
1.4	Sex	Code: 1 = Male, 2 = Female	|___|
1.5	Marital status (for 12 yrs old and above)	Code: 1 = Never married, 2 = Married, 3 = Divorced<br>4 = Separated, 5 = Widowed	|___|
1.6	Religion	Codes: 1 = Islam, 2 = Hindu, 3 = Buddhist, 4 = Christianity,<br>5 = Others (specify) ____	|___|
1.7	Municipality/Pourasava of residence	Yes = 01, No = 02, Don't know = 03	|___|___|
1.8	Number of years of schooling completed (passed)		|___|___|
1.9	Was s/he continuing the schooling?	Yes = 01, No = 02	|___|___|
1.10	Occupation at time of death (most recent occupation) for deceased more than 10 years old, please specify ____________________________		|___|___|___|

	If deceased was <12 yrs old	Mother	Father
1.11	Name/ CID		
1.12	Date of birth		
1.13	Education (class passed)		
1.14	HDSS occupation and code	|___|___|___|	|___|___|___|
1.15	Marital status<br>Codes: 1 = Never married, 2 = Married, 3 = Divorced, 4 = Separated, 5 = Widowed		|___|

2. INFORMATION ABOUT THE HOUSEHOLD HEAD

2.1	Name of the HH head & CID		CID:
2.2	Date of Birth: Age _______years		|___||___| |___||___| |___||___||___||___| dd mm yyyy
2.3	Sex	Code: 1 = Male, 2 = Female	|___|
2.4	Marital status (for 12 yrs old and above)	Code: 1 = Never married, 2 = Married<br>3 = Divorced, 4 = Separated, 5= Widowed	|___|
2.5	Education (class passed) _________________________________		|___||___|
2.6	HDSS occupation and code __________________________		|___|___|___|
2.7	Does any household member have a health insurance?		|___| 1=Yes |___| 2= No
2.8	If yes, specify the insurance …………………………………		

3. TRAVEL TIME TO UPAZILA HEALTH COMPLEX (UHC)/DISTRICT HOSPITAL (DH) / PRIVATE CLINIC (PC)/ OTHER HOSPITAL (OH), FOR HOSPITAL DEATH ONLY

3.1	Distance of residence at time of terminal illness to UHC/DH / PC/ OH in kilometers (Information on distance will be validated from existing information)		|_1__| |_2__| |_3__| |_4 _| UHC DH PC OH<br>Km |___||___||___|
3.2	Did you bring the deceased directly to UHC/DH/ PC/ OH?	|___| 1 Yes CONTINUE<br>|___| 2 No SKIP TO 3.5	|___|
3.3	If Yes, how long did you have to travel from your house to UHC/DH/PC/OH in minutes?	VERBATIM ________________	|___||___||___|
3.4	How much did you spend to travel to UHC/DH /PC/OH in taka?<br>**TERMINATE INTERVIEW**	VERBATIM ________________	|___||___||___||___|
3.5	If NO, to which hospital, health facility or physician, did you bring her/him to **FIRST** before s/he was moved to UHC/DH/ PC/ OH	VERBATIM<br>________________________	|___||___|
3.6	How long did you have to travel from your house to this facility in minutes?	VERBATIM ________________	|___||___||___|
3.7	How much did it cost you to travel from your house to this facility in taka?	VERBATIM ________________	|___||___||___||___|
3.8	How long did you have to travel from this facility to UHC/DH/ PC/ OH in minutes?	VERBATIM ________________	|___||___||___|
3.9	How much did it cost you to travel from this facility to UHC/DH/ PC/ OH in taka?	VERBATIM ________________	|___||___||___||___|

4. HOUSEHOLD ASSETS

4.1	Land own (in decimal)	Homestead<br>|___||___||___||___|	Agriculture and others<br>|___||___||___||___|	Total<br>|___||___||___||___|
4.2	Number of HH members			|___|___|
4.3	Number of sleeping rooms			|___|
4.4	Main source of drinking water	01 Tube well (green)<br>02 Tube well (red)<br>03 Tube well (not yet tested)<br>04 Deep tube well (600+ feet)<br>05 Pond / River/Ditch/Canal<br>06 PSF/RSF (Pond Sand Filter/River Sand Filter)<br>07 Three pitchers<br>08 Rain water<br>09 Other water filters<br>10 Piped water from own house<br>11 Piped water from neighbor/ public taps<br>12 Others (specify)		|__|__|

4.5	Toilet type used by females	01 Sanitary latrine<br>02 Ring/ Slab but waste not drained out<br>03 Ring/ Slab but waste drained out<br>04 *Pacca* latrine but waste drained out<br>05 *Kancha* latrine<br>06 Other (specify)	|__|__|
4.6	Fuel used for cooking	01 Gas<br>02 Firewood/straw/charcoal<br>03 Cow dung/leaves<br>04 LPG gas<br>05 Electricity<br>06 Kerosine<br>07 Other	|__|__|
4.7	Housing materials (dwelling):	01 Earth/Mud<br>02 Cement/Tiles<br>03 Tin<br>04 Thatch/Straw/Leaves<br>05 Scrap materials/Sack/Polythine<br>06 Wood<br>07 Bamboo<br>08 Other, specify ……….………	|__|__| |__|__| |__|__| Roof Wall Floor

		1 = Yes<br>2 = No			1 = Yes<br>2 = No
4.8	Separate cooking room ?		4.26	Own pump for irrigation?	
4.9	Have lantern (*Hurricane*)?		4.27	Own thresher machine?	
4.10	Have quilt/blanket?		4.28	Have solar panel (connection)?	
4.11	Have mattress?		4.29	Own a sewing machine?	
4.12	Have bed stand (khat/chowki)?		4.30	Own a CD/VCD/DVD player?	
4.13	Have chair/table?		4.31	Own landline phone?	
4.14	Have rickshaw/rickshaw van?		4.32	Own a country boat	
4.15	Own bicycle?		4.33	Own a motorboat?	
4.16	Own mobile phone?		4.34	Have fishing net?	
4.17	Have dining table?		4.35	Have chicken/duck? (mention #)	
4.18	Have showcase/almirah?		4.36	Have cow/goat? (mention #)	
4.19	Have sofa set?		4.37	Own a shop?	
4.20	Have electricity?		4.38	Own fridge/refrigerator?	
4.21	Have electric fan?		4.39	Own a motorcycle?	
4.22	Own radio?		4.40	IPS (Instant Power Supply)	
4.23	Own television?		4.41	Own a computer?	
4.24	Auto rickshaw: CNG/ Battery/Petrol		4.42	Own a car?	
4.25	Own power tiller?		4.43	Generator	

Name of Interviewer: ________________________________ Code |___|___|___|

Date of Household Visit |___||___| |___||___| |___||___||___||___|
dd mm yyyy
