# Supplementary figures and images for: On the estimation of population cause-specific mortality fractions from in-hospital deaths
Source: BMC Med. 2019 Feb 8;17:29. doi: 10.1186/s12916-019-1267-z (PMC6367755; doi:10.1186/s12916-019-1267-z)

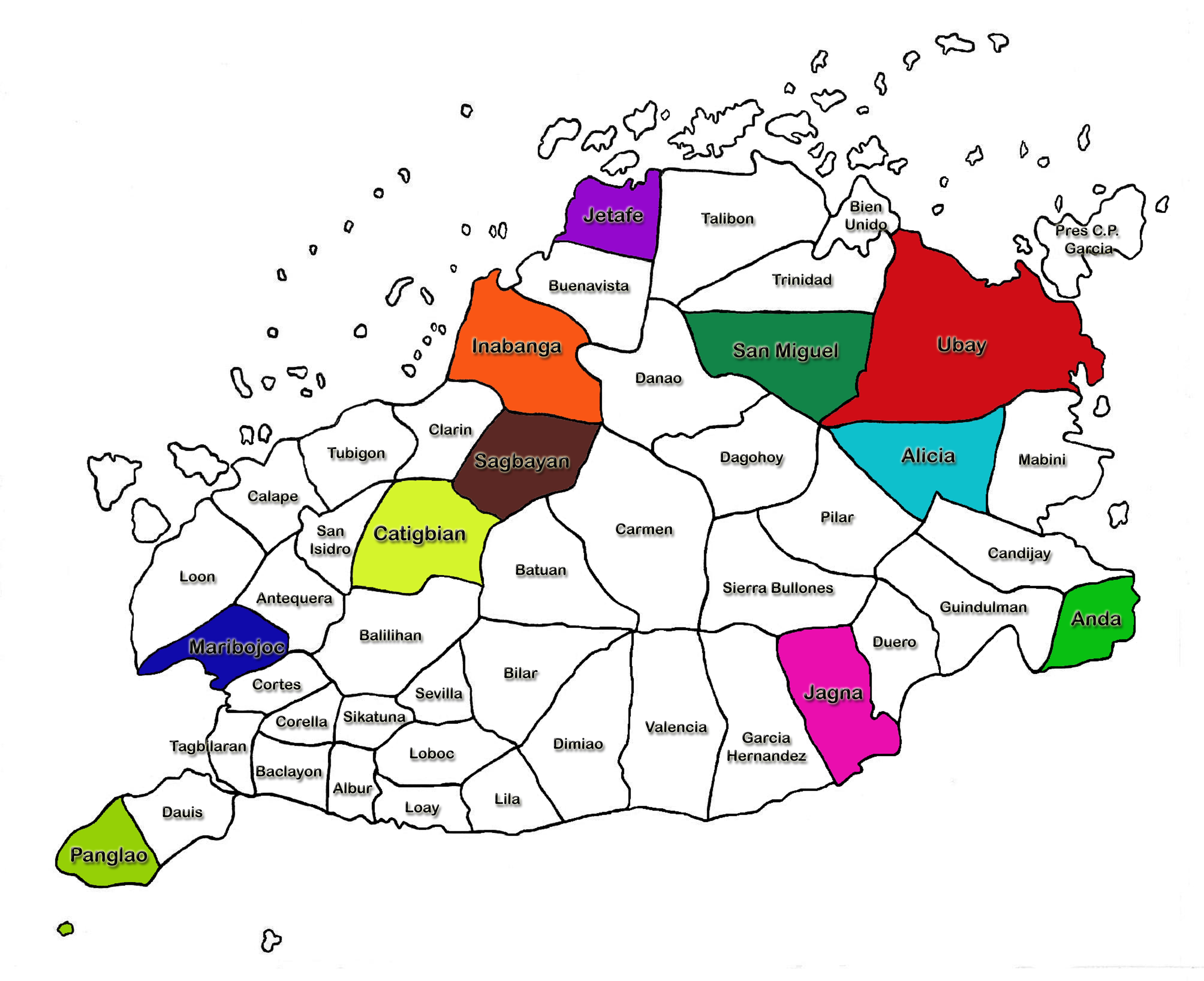

Supplement: Supplementary file 3 — Map of Bohol, Philippines. Map of hospital catchment areas in Bohol. (PNG 6975 kb) [file 12916_2019_1267_MOESM3_ESM.png]

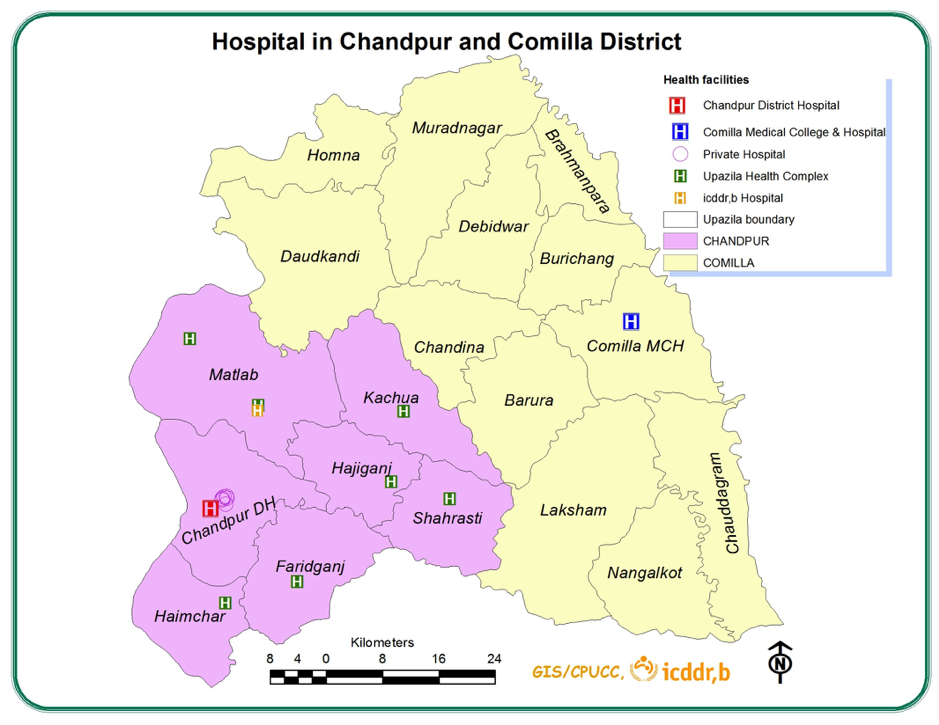

Supplement: Supplementary file 4 — Map of Bangladesh. Map of hospital catchment areas in Bangladesh. (PNG 404 kb) [file 12916_2019_1267_MOESM4_ESM.png]
