# Supplementary material for: On the estimation of population cause-specific mortality fractions from in-hospital deaths
Source: BMC Med. 2019 Feb 8;17:29. doi: 10.1186/s12916-019-1267-z (PMC6367755; doi:10.1186/s12916-019-1267-z)
Supplement: Supplementary file 6 — Death per hospital death model appendix. Additional information on calculating the point estimates and confidence intervals for the deaths per hospital death model. (DOCX 16 kb) [file 12916_2019_1267_MOESM6_ESM.docx]

**Method for obtaining CSMF and CIs using the death per hospital death (DPHD) method**

A ‘crude’ CSMF can be estimated from the Population Sample alone, by estimating the proportion within each cause of death (COD) group. Standard errors and 95% confidence intervals (Cis) can be calculated using the usual formula for an estimator of a proportion based on a multinomial distribution.

A slightly more refined estimate can be obtained by estimating the probability of death in hospital, for each COD group separately, inverting this to get the total expected number of deaths in the population associated with a single death in hospital. This is then multiplied by the number of hospital deaths in the COD group to get the imputed number of deaths in that COD group in the population.

If pi is the proportion of deaths in hospital for the ith COD, estimated from the Population Sample, and Ni is the total number of deaths in the ith COD, based on the Hospital Sample, then

Hi = Ni/pi

is the imputed number of deaths in the population in the ith COD group. The CSMF may be estimated by calculating the proportion of imputed deaths in each COD, out of the overall total of all imputed deaths.

For the purpose of deriving a formula for a confidence interval for CSMF it is preferable to log-transform the CSMF first and derive a standard error formula for log(CSMF) as a Taylor-series expansion of the log(CSMF) estimator, assuming that the total number of hospital deaths is Poisson distributed, and that the proportion in each COD group is multinomially distributed. This yields

SE (log(CSMF)) = $\surd(\frac{1}{N_{i}}+\frac{1-p_{i}}{M_{i}p_{i}}$ )

where Mi is the total number of deaths in the Population Sample in the ith COD.

Logistic regression produces linear predictions for selected subgroup of Hospital Deaths. These are in the k x 1 vector:

l = X’ β

where X is a k x (q+1) matrix containing the q covariate values for the k selected Hospital deaths, with the first column containing 1s to correspond to the intercept parameter and β is a (q+1) x 1 vector containing the q+1 coefficients from the logistic regression. The elements of l are equivalent to log(p_i_/(1-p_i_)), where p_i_ is the predicted probability in hospital for the ith Hospital death.

The variance-covariance matric for l = X’ β is

Cov(l) = X’ Cov(β) X.

An estimate of Cov(β) is given by PROC LOGISTIC as output.

The predicted number of total deaths, corresponding to each Hospital death is

H_i_ = 1/p_i_ = 1 + exp(-l_i_)

and the total number of predicted deaths is

T = I’ H

where I is a k x 1 vector of 1s and H is the k x 1 vector containing the H_i_.

The variance of T, Var(T), is

I’ Cov(H) I

where Cov(H) is the variance-covariance matrix for H.

The elements of Cov(H) are

Cov(H_i_, H_j_) = (∂H_i_/∂l_i_ ∂H_j_/∂l_j_) Cov(l_i_, l_j_)

= exp(-l_i_) exp(-l_j_) Cov(l_i_, l_j_)

= (H_i_ - 1) (H_j_ -1) Cov(l_i_, l_j_)

For a subgroup C of deaths, T = T_C_ and Var(T_C_) = V(T_C_).

Adding over subgroups gives ΣT = ST and V(ST) = Σ_d_V(T_d_).

Var( log (T_C_/ST) ) = Var( log (T_C_) – log(ST) )

= (1/T_C_)^2^V(T_C_) + (1/ST)^2^V(ST) – 2( 1/(T_C_ST) )V(T_C_)

A 95% CI for CSMF = T_C_/ST is then

CSMF/ exp(1.96 sqrt(Var( log (T_C_/ST) ) ) ), CSMF exp(1.96 sqrt(Var( log (T_C_/ST) ) ) )

Detailed programming steps:

For each subgroup:

1. Run PROC LOGISTIC to get β and Cov(β) – store in datasets B and CovB respectively. Store l in dataset L as check on later calculations.
2. Create dataset corresponding to X.
3. Import B, CovB and X into IML as matrices. Create I as a matrix.
4. Calculate l, Cov(l), H, T, Cov(H) and Var(T) in IML
5. Export T and Var(T) to SAS datasets
6. Consolidate T and Var(T) in a single dataset. Get TotalT and Var(TotalT). Calculate CSMF and 95% CI
7. Print T, CSMF with 95% CIs for each subgroup to HTML file in SAS.

The matrix manipulations described above were performed using SAS IML.

Models were compared using the AIC criteria. Sensitivity analyses examined robustness of the model when COD categories were small.

This method can result in very small predicted probabilities of death in hospital, depending on the distributions of covariates and their association with death in hospital. This may lead to extreme values for the number of impute deaths. To protect against unduly influential outliers in this process, the distributions of H were Winsorised. This is a process in which values above a selected percentile are set equal to the percentile of the distribution. The 99^th^ percentile was selected for this process.

Cross-validation was used to examine to examine the robustness of the final estimates.
